# Cross-cultural adaptation and validation of the caregiver self-efficacy in contributing to patient self-care scale in China

**DOI:** 10.1186/s12889-024-19534-2

**Published:** 2024-07-24

**Authors:** Qingyun Lv, Xiaonan Zhang, Yaqi Wang, Xueying Xu, Xiaoying Zang

**Affiliations:** Tianjin Med Univ, Sch Nursing, 22 Qixiangtai Rd, Tianjin, 300070 P. R. China

**Keywords:** Multiple chronic conditions, Caregiver self-efficacy, Rasch analysis, Reliability, Validity

## Abstract

**Background:**

Caregiver self-efficacy is crucial in improving patient outcomes and caregiver well-being, but there is a lack of suitable scales to assess this concept within the context of Chinese culture. This study aimed to cross-culturally translate the Caregiver Self-Efficacy in Contributing to Patient Self-Care (CSE-CSC) Scale and evaluate its psychometric properties using classical test theory and item response theory.

**Methods:**

The CSE-CSC scale was adapted using Brislin’s translation model after obtaining authorization from the original author. A multicenter, cross-sectional study was conducted to assess the psychometric properties of this scale. Classical test theory was used to evaluate reliability (internal consistency, test-retest reliability), validity (content validity, structural validity, convergent validity), and floor and ceiling effects. Item response theory was employed to assess the fit of the rating scale model, reliability, item difficulties, and measurement invariance.

**Results:**

The translation and cultural adaptation process was completed. Classical test theory demonstrated good internal consistency (Cronbach’s α = 0.935) and test-retest reliability (ICC from 0.784 to 0.829, *p*<0.001). The I-CVI and K* of each item ranged from 0.875 to 1.00 and 0.871 to 1.00. The first-order 2-factor model fit well (*χ*^2^/*df* = 3.71, RMSEA = 0.082, SRMR = 0.032, CFI = 0.973, TLI = 0.60). Convergent validity showed that the CSE-CSC scores had a strong positive correlation with three separate scales of the CC-SC-CII. There was no floor and ceiling effect in this scale. Rasch analysis showed that the CSE-CSC scale demonstrated a good fit to the rating scale model and exhibited excellent reliability (person/item separation index>2, person/item reliability coefficients>0.8). The Wright map showed that item difficulty matched the respondents’ measured abilities. The analysis of differential item functioning (DIF) showed that all items were comparable in gender.

**Conclusions:**

This study indicated that the CSE-CSC scale had good reliability, validity, difficulty degree, and measurement invariance. The CSE-CSC scale can be used to measure caregiver self-efficacy of Chinese patients with multiple chronic conditions.

## Background

Individuals with multiple chronic conditions (MCCs) are defined as patients with two or more concurrent chronic behavioral or physical conditions [1]. It has been estimated that more than 20% of adults globally suffer from MCCs [2]. MCCs are more common in the older population compared with the general population. The prevalence of MCCs in older people over 65 years reported by different studies ranged from 55 to 98% [2]. In China, the prevalence of MCCs reached 49% in community-dwelling older adults [3]. MCCs affect each other synergistically, leading to a more pronounced impact on patients’ health-related quality of life, physical limitations, and mortality [4, 5]. Additionally, chronic conditions usually require ongoing medical attention and support, resulting in a greater utilization of medical resources by individuals with MCCs. About 31% of the population with 1 or 2 conditions drove 23% of overall healthcare spending, while about 12% of the population with 5 or more chronic conditions was responsible for 41% of spending [1]. Self-care, defined as the process of maintaining one’s health through health-promoting behaviors and disease management [6], has been proven to be critical for improving clinical outcomes and reducing financial burden in MCCs [7]. Self-care in patients with MCCs includes prioritizing ever-changing needs, conditions, and goals, which defined as a dyadic phenomenon [8, 9]. Patients with MCCs are often older and have difficulty in integrating self-care across conditions, so a qualified caregiver is crucial to support patient self-care [10, 11].

Caregiver self-efficacy has been defined as the belief of caregivers in their ability to assist patients with self-care, including helping patients maintain disease stability, promoting symptom monitoring and perception, and responding to elevated physical conditions [12]. The caregiver self-efficacy can enhance patient adherence to disease management and subsequently have a positive impact on patient outcomes [13]. The evidence suggests that high levels of self-efficacy among caregivers could improve the functional and physical well-being of lung cancer patients and alleviate the severity of depression symptom [14]. Moreover, a survey conducted on stroke patients revealed that the self-efficacy level of partner who served as informal caregiver was significantly associated with the patient’s depression level and life satisfaction [15]. Besides, caregiver self-efficacy plays an important role in improving caregiver well-being. A previous study indicated that caregiver self-efficacy was associated with caregiver depression and burden [16].

Although caregiver self-efficacy has been associated with positive health outcomes for patients with MCCs and their caregivers, there are challenges in measuring caregiver self-efficacy for this specific population. The General Self-Efficacy Scale is usually used to evaluate belief in one’s competence to cope with a broad range of stressful or challenging demands, which may not fully capture the specific challenges faced by caregivers of patients with MCCs [17]. In addition, some tools for measuring caregiver self-efficacy have only been explored in the context of single chronic conditions, such as the Caregiver Caregiving Self-Efficacy Scale-Oral Cancer [18], and the Caregiver Confidence in Sign/Symptom Management scale [19]. The 10-item CSE-CSC scale was proposed based on the theory of Self-Care of Chronic Illness to measure caregiver self-efficacy in contributing to patient self-care maintenance, monitoring, and management of multiple chronic illnesses [20]. However, the CSE-CSC scale has not been translated into Chinese and validated.

There are two primary methodologies for evaluating the psychometric properties of a scale: classical test theory (CTT) and item response theory (IRT). While CTT has been extensively utilized for assessing instrument psychometric properties, it is important to acknowledge its limitations, such as its reliance on the sample and the assumption that each item contributes equally to the total scores [21]. IRT offers solutions to these limitations by considering the probability of a correct response based on individual abilities and item characteristics, enabling a more nuanced examination of item properties like difficulty and discrimination [22]. Integrating CTT and IRT can provide a comprehensive assessment of a scale’s properties, addressing the limitations of each approach [23]. Therefore, our study aimed to translate the CSE-CSC into Chinese and evaluate its psychometric properties based on CTT and IRT.

## Methods

### Participants and settings

From July 2022 to July 2023, the recruitment of caregivers was completed in the cardiology departments of 4 third-class hospitals in Tianjin, China. The inclusion criteria of caregivers were: (a) aged 18 years or older; (b) family members or close relatives of the patients, providing majority informal care tasks (e.g., help in daily activities); (c) provided care for patients who were aged 18 years or older, diagnosed with multiple chronic conditions according to Charlson Comorbidity Index [24]; (d) had clear awareness, reading and language expression ability, independent response and accessible communication; and (e) informed consent for both caregiver and patients. In our study, we excluded caregivers who were paid and had participated in the intervention trial for nearly three months.

The sample size was determined based on a subject-to-item ratio of 10:1 by assuming a non-response rate of 15% [25]. Thus, the sample size was at least 118. Additionally, large samples, usually 300 participants, are necessary for a Rasch model to obtain robust parameter estimates [26]. Finally, we enrolled 406 participants to support stable analysis.

### Design and procedures

**Fig. 1:**
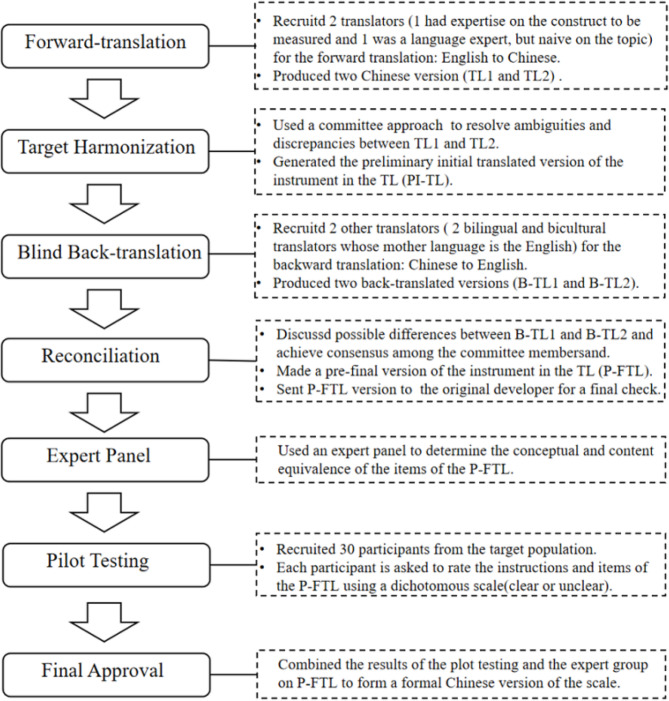
The cross-cultural translation and cultural debugging of the CSE-CSC scale

The translation and cross-cultural adaptation of the English CSE-CSC scale were conducted after obtaining written permission from Professor Maddalena De Maria, the developer of the original scale. The translation was based on Brislin’s translation model and included four steps: forward-translation, target harmonization, blind back-translation, and reconciliation [27]. These steps were repeated in cycles. If ambiguities and discrepancies could not be resolved, the process was repeated as necessary. Cross-cultural debugging was carried out through expert consultation and pilot testing to form the final version (Fig. 1).

### Data collection

One data collector was selected from each of the four hospitals and received uniform training to minimize survey and measurement bias. The study purpose and data collection procedure were explained by the data collectors before the survey began. Under the uniform guidance of trained data collectors, 406 participants who provided informed consent were surveyed. Specifically, 61 participants were recruited from the first hospital, 115 from the second hospital, 179 from the third hospital, and 51 from the last hospital. A total of 30 participants who voluntarily left contact information underwent a remeasurement after a 2-week interval. Participants were given the choice to complete the questionnaires online or on paper. The effective recovery rate of the questionnaire was 100%.

### Measures

1. Sociodemographic questionnaire: This part was self-designed and included sociodemographic characteristics of the caregivers and their patients, such as age, gender, education, work status, years of caregiving.
2. The Caregiver Self-Efficacy in Contributing to Patient Self-Care (CSE-CSC) Scale: The CSE-CSC scale is a 10-item scale produced by Maddalena De Maria et al. [20] and includes two subscales: Self-efficacy in self-care maintenance and monitoring, Self-efficacy in self-care management. The CSE-CSC scale uses a 5-point Likert format (1 = “not confident” to 5 = “very confident”), and the total score is standardized on a scale of 0 to 100, where higher scores mean higher caregiver self-efficacy in contributing to the self-care of patients with MCCs. The Cronbach’s alpha value of the original version was 0.942 for the whole scale [20].
3. The Caregiver Contribution to Self-Care of Chronic Illness Inventory (CC-SC-CII): The CC-SC-CII is a 19-item tool that measures informal caregiver contribution to self-care behaviors in patients with chronic illness. It consists of three separate scales: the caregiver contribution to self-care maintenance scale, the caregiver contribution to self-care monitoring scale, and the caregiver contribution to self-care management scale [28]. Each scale has a score range of 0 to 100, with higher scores indicating better caregiver contribution. The Chinese version of CC-SC-CII was translated by Dandan Chen, and the Cronbach’s alpha values of three scales were 0.792, 0.880, and 0.870 [29].

### Data analysis

Data analysis was conducted using SPSS 24.0, Mplus 8.3, and WINSTEPS 3.6.6, and a *p*-value < 0.05 was considered significant.

#### Validity and reliability testing using CTT

1. Content validity: Content validity index (CVI) and Kappa value (K*) were used to evaluate content validity. Experts were asked to fill out a 4-level content equivalence scale (1 = “not relevant” to 4 = “very relevant and succinct”). The inclusion criteria for experts were as follows: (1) having rich experience in chronic disease management; (2) having been working at least for ten years; (3) being willing to participate in this research. I-CVI was calculated by dividing the number of experts with a relevance rating of 3 or 4 by the total number of experts. S-CVI was calculated by the averaging method, which is the mean value of I-CVI for each item [30]. To avoid the randomness of expert opinion, each item’s Kappa value (K*) was calculated by SPSS 24.0. Quality criteria of good content validity: I-CVI ≥ 0.78, S-CVI/Ave ≥ 0.90, and K*≥ 0.60 [31].
2. Structural validity: Confirmatory factor analysis (CFA) was performed to assess the structural validity. The internal structure of the CSE-CSC scale was evaluated using the maximum likelihood estimation method. Overall fit indexes were employed to identify the model’s goodness: normed χ^2^/*df* < 3.0, the root mean square error of approximation (RMSEA) < 0.08, the standardized root mean square residual (SRMR)<0.08, the Comparative Fit Index (CFI) and Tucker-Lewis Index (TLI) > 0.9 [32]. The model’s misfit was improved by considering the eventual residual covariances justified to theoretical and methodological reasons.
3. Convergent validity: Convergent validity was tested by examining the correlation between the CSE-CSC scale and CC-SC-CII scale scores. The hypothesis was that the CSE-CSC scale would have a large positive correlation (Pearson correlation coefficient *r* ≥ 0.50) with the CC-SC-CII, as reported in the theory [12] and previous study [20].
4. Reliability: Reliability was evaluated using internal consistency and test-retest reliability. A Cronbach’s α of ≥ 0.70 indicated high internal consistency for the scale. Test-retest reliability means stability, reliability, or reproducibility among the same group of respondents between two time points. In the study, 30 participants were remeasured after two weeks [33]. Intraclass correlation coefficients (ICC) were used to identify test-retest reliability, with quality criteria of ICC ≥ 0.70 for good test-retest reliability [34].
5. Floor/ceiling effect: Floor effects were evaluated by examining the percentage of the respondents who achieved the lowest possible scores, while ceiling effects were assessed by looking at the percentage of respondents who reached the highest possible score. If more than 15% of respondents achieved the highest or lowest possible score, it indicates a ceiling or floor effect [34].

#### Validity and reliability testing using IRT

1. Structural validity: The structural validity using Rasch analysis can be rated as sufficient when assumptions on monotonicity, unidimensionality, and local independence are not violated and when there is an adequate model fit [35]. Monotonicity means that the probability of a “correct” response cannot fluctuate, which can be determined based on the item characteristic curves [36]. Principal component analysis was used to test unidimensionality. If the Rasch model explains at least 40% of the variance and the eigenvalue of “Unexplained variance in 1st contrast” does not exceed 3, the scale meets the assumption of unidimensionality [37]. Local independence means that responses to an item should not be dependent on responses to other items. After controlling for the dominant factor, this is violated when residual correlations among the items were greater than 0.3 [38]. The overall fit degree to the Rasch model was estimated by Infit MNSQ and Outfit MNSQ, where a range of 0.5 to 1.5 means fit to the Rasch model [35]. If the result is beyond this range, consideration should be given to whether to carry out item censoring or refinement. Positive and high values (> 0.3) of point-measure correlation indicated that the items were working in the same direction to measure a single basic construct [39].
2. Reliability analysis: The indicators used to assess reliability were the Person/Item separation index and Person/Item reliability coefficients. Separation indices were employed to assess the difficulty of a project or the continuous distribution of an individual’s ability, and scores exceeding 2.0 indicated a good separation level. Reliability coefficients gauged the replicability of the measurement results, and scores surpassing 0.8 signified good reliability [39].
3. Item difficulties: Item measure meant the difficulty level of the item for the subjects, with higher values indicating greater difficulty for the item. Besides, a Wright map was used to visually compare the difficulty of the scale items with the participants’ abilities. The Wright map directly compared item difficulty and respondents’ measured abilities in one logit Rasch “bar”.
4. Measurement invariance: To examine whether the scale items were used in the same way by all groups, a differential item functioning (DIF) was conducted. Logit scores were compared for each item between different genders using the Welch t-test to assess DIF. DIF should be noticed when item DIF Prob. < 0.05 logits [40].

### Ethical considerations

The research was approved by the Ethics Committee of Tianjin Medical University (Grant number: TMuhMEC2022021). Both caregivers and patients provided written informed consent for participating in the study.

## Results

### Translation and cultural adaptation

The CSE-CSC scale was translated strictly following the Brislin’s translation model. In order to make the scale easier to accept and understand by the caregivers in the Chinese cultural context and also identify the clarity of the instructions, conceptual and content equivalence, a two-round expert consultation was conducted. The scale was modified in three aspects: (1) The “the person you care for” of the original scale was changed to “patient” to enhance clarity for caregivers, which will improve caregivers’ response speed. (2) Item 2 in the original scale, “Follow the treatment plan that has been given to the person you care for?” did not specify the treatment makers, leading to confusion among caregivers. Experts observed that within the Chinese cultural context, caregivers may sometimes adopt health management methods from unofficial healthcare organizations. Therefore, it is necessary to clarify that the treatment plan refers specifically to those prescribed by medical professionals. (3) The “conditions” was changed to “health conditions” in items 4 and 5. During the plot testing, all 10 participants thought all the items were well-articulated, and the meaning was easily understood. The revised scale used for the final population validation was consistent with the original intention of the scale developers and the Chinese culture. The original CSE-CSC scale and its translated Chinese version are presented in Table 1.

**Table 1:** The items in original CSE-CSC scale and its Chinese version

Item	Original English version	Target Chinese version
1	Keep the illness of the person you care for stable and free of symptoms?	保持患者病情稳定且没有症状
2	Follow the treatment plan that has been given to the person you care for?	遵从医护人员为患者制定的治疗方案
3	Persist in following the treatment plan even when difficult?	在有困难时依然坚持遵从治疗方案
4	Routinely monitor the condition of the person you care for?	定期监测患者的身体状况
5	Persist in routinely monitoring the condition of the person you care for even when difficult?	在有困难时依然坚持定期监测患者的身体状况
6	Recognize changes in the health of the person you care for if they occur?	意识到患者的健康状况出现变化
7	Evaluate the importance of symptoms?	评估患者的症状是否重要
8	Do something to relieve symptoms of the person you care for?	采取一些措施来缓解患者的症状
9	Persist in finding a remedy for symptoms of the person you care for even when difficult?	在有困难时依然坚持寻找可以缓解患者症状的治疗方法措施?
10	Evaluate how well a remedy works?	判断缓解症状的方法措施是否有效

### Participants characteristics

Table 2 presents the demographic characteristics of the 406 caregivers and their patients. The caregivers had an average age of 56.35 ± 14.265 years, with 252 (62.1%) female. Care tasks were predominantly undertaken by spouses/partners or children, covering 95.3% of the patients. Half of the caregivers provided care to patients for more than six years. About 58.9% of the patients were male, and 52.2% had 2–4 chronic diseases.

**Table 2:** Clinical and sociodemographic characteristics of caregivers and patients (*N* = 406)

Characteristics	Caregiver (*N*/ %)	Patient (*N*/ %)
Age (M ± SD)	56.35 ± 14.265	71.22 ± 11.929
Gender		
Male	154 (37.9)	239 (58.9)
Female	252 (62.1)	167 (41.1)
Residential location		
Urban	314 (77.3)	-
Town/countryside	92 (22.7)	-
Education level		
Primary school or less	58 (14.3)	-
Middle school	161 (39.7)	-
High school	150 (36.9)	-
College/university degree or above	37 (9.1)	-
Relationship with patient		
Spouse/partner	190 (46.8)	-
Child	197 (48.5)	-
parents	4 (1.0)	-
Sister/brother/friend	15 (3.7)	-
Living with patient	348 (85.7)	-
Times providing care to patient		
<3y	61 (15.0)	-
3-6y	142 (35.0)	-
>6y	203 (50.0)	-
Work status		
Manual work^1^	151 (37.2)	-
Brain work^2^	79 (19.5)	-
Retirement	114 (28.1)	-
No work	62 (15.3)	-
Economic status (yuan/month/per individual)		
<¥3000	100 (24.6)	-
¥3000–6000	193 (47.5)	-
>¥6000	113 (27.8)	-
Current marital status		
Single	11 (2.7)	-
Married or have a partner	381 (93.8)	-
Divorced or widowed	14 (3.5)	-
Number of chronic illnesses		
2–4	-	212 (52.2)
5–7	-	160 (39.4)
≥ 8	-	34 (8.4)

^1^included professional and technical work such as administrative personnel, salespeople, and office staff; ^2^included tasks primarily requiring physical exertion, such as agricultural production, craftsmen, and workers in the service industry

### Validity and reliability testing using CTT

#### Content validity

The results of expert consultation, consisting of eight experts, showed that the I-CVI of the CSE-CSC scale ranged from 0.875 to 1.00, with an overall S-CVI of 0.95. K* values for each item ranged from 0.871 to 1.00. These findings indicated that the content validity of the CSE-CSC was good.

#### Structural validity

**Fig. 2:**
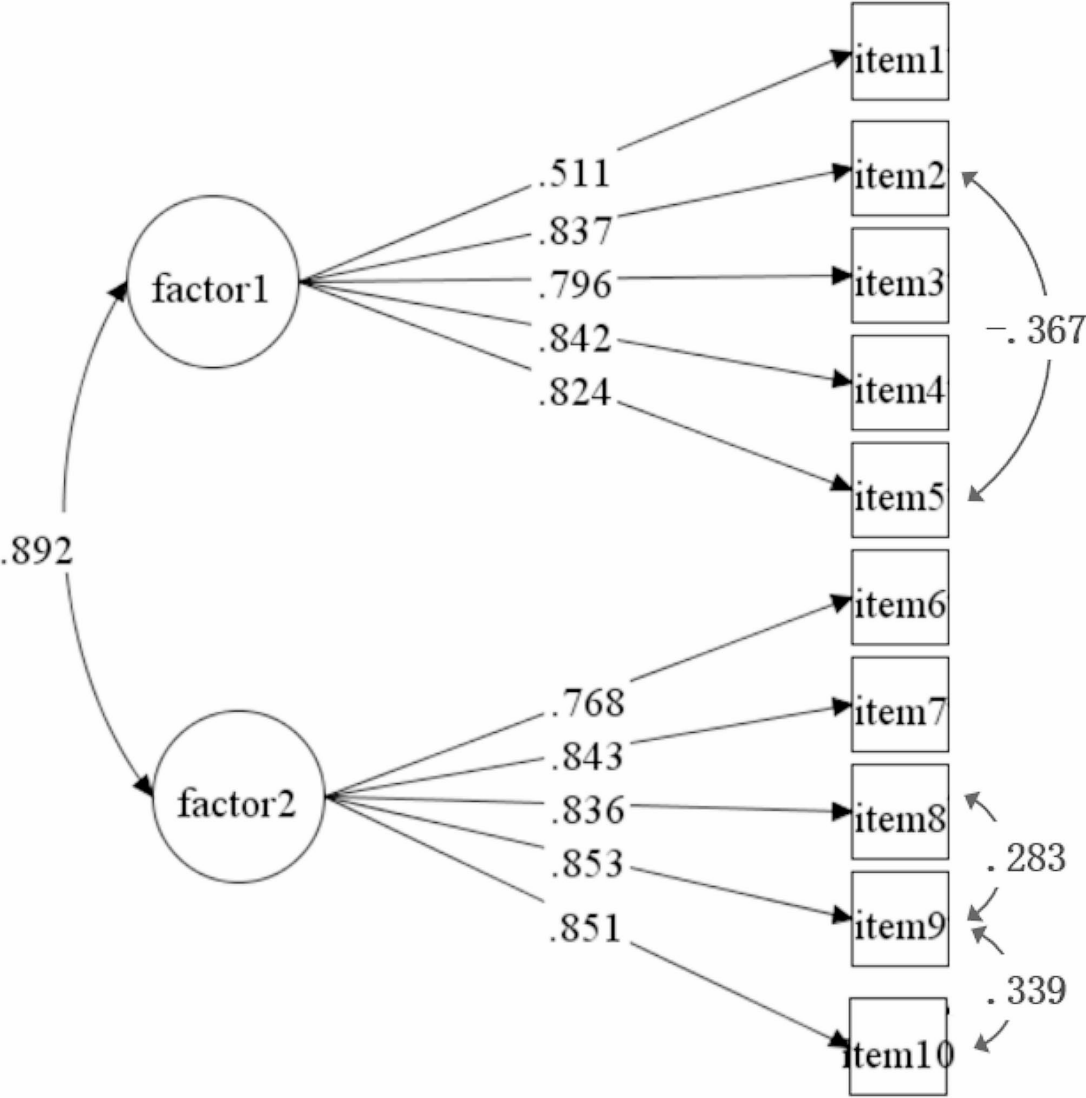
The first-order 2-factor model in Confirmatory factor analysis of the Chinese version CSE-CSC scale

The results of CFA showed that the second-order 2-factor structure supported by the original scale did not exhibit a good fit in this study (see row 1 of Table 3). Based on the original structure, the first-order 2-factor and first-order 1-factor models were verified using the same samples. The first-order 2-factor model demonstrated the best overall fit indexes, with RMSEA = 0.082, SRMR = 0.032, CFI = 0.973, and TLI = 0.60. The *χ*^2^/*df* more than 3 may be due to the lack of a sufficiently large sample size [41]. Factor loading of each item ranged from 0.511 to 0.853, as illustrated in Fig. 2. The model fit was improved by estimating residual covariances between three pairs of items: item 2 and item 5, item 8 and item 9, item 9 and item 10. Item 2 and item 5 were the self-efficacy in self-care maintenance and monitoring dimension. Item 8, 9, and 10 focused on symptom management. The covariances between item residuals were allowed because they followed principles of reasonable methodology or theory [20].

**Table 3:** Fit indices of the Chinese version CSE-CSC scale from CFA

Statistical Model	χ^2^	df	χ^2^/df	TLI	CFI	SRMR	RMSEA (90% CI)
The second-order 2-factor model	186.525	33	5.652	0.932	0.950	0.038	0.107(0.092,0.122)
The first-order 2-factor model	115.143	31	3.714	0.960	0.973	0.032	0.082(0.066,0.089)
The first-order 1-factor model	175.697	32	5.490	0.934	0.953	0.038	0.105(0.090,0.121)

#### Convergent validity

The results of Pearson’s correlation analysis showed that the CSE-CSC scores had strong positive correlations with three separate scales of the CC-SC-CII: *r* = 0.605 (*P*<0.001), *r* = 0.541 (*P*<0.001), and *r* = 0.563(*P*<0.001), respectively.

#### Reliability

The Cronbach’s alpha coefficients of the total scale and two factors were 0.935, 0.863, and 0.963, respectively. Although Cronbach’s alpha of total scale and factor 2 were both greater than 0.9, indicating a potential issue of item redundancy, considering the clinical significance of the scale items and the theory of self-efficacy, the internal consistency of the scale was still recognized in this study. The CSE-CSC and two factors had good test-retest reliability, and the ICCs were 0.810 (*P*<0.001), 0.784 (*P*<0.001), and 0.829 (*P*<0.001), respectively.

#### Floor/ceiling effect

The theoretical and practical score ranges of the CSE-CSC scale were presented in Table 4. The biggest percentage of the occurrences of the lowest/highest possible was 5.17%, so there was no floor and ceiling effect in this scale and two dimensions, which indicated that the Chinese version scale can adequately reflect the actual level of caregiver self-efficacy in contributing to patient self-care.

**Table 4:** The floor/ceiling effect analysis of the CSE-CSC scale and two subscales

Scale/Subscale	Score ranges		Lowest score (%)	Highest score (%)
	Theoretical	Practical		
Subscale				
Self-efficacy in self-care maintenance and monitoring	5–25	5–24	0.25% (1/406)	0.00% (0/406)
Self-efficacy in self-care management	5–25	5–25	0.25% (1/406)	5.17% (21/406)
the CSE-CSC scale	0-100	0-97.50	0.25% (1/406)	0.00% (0/406)

### Rasch analysis of the CSE-CSC

#### Unidimensionality, local dependency, and monotonicity

**Fig. 3:**
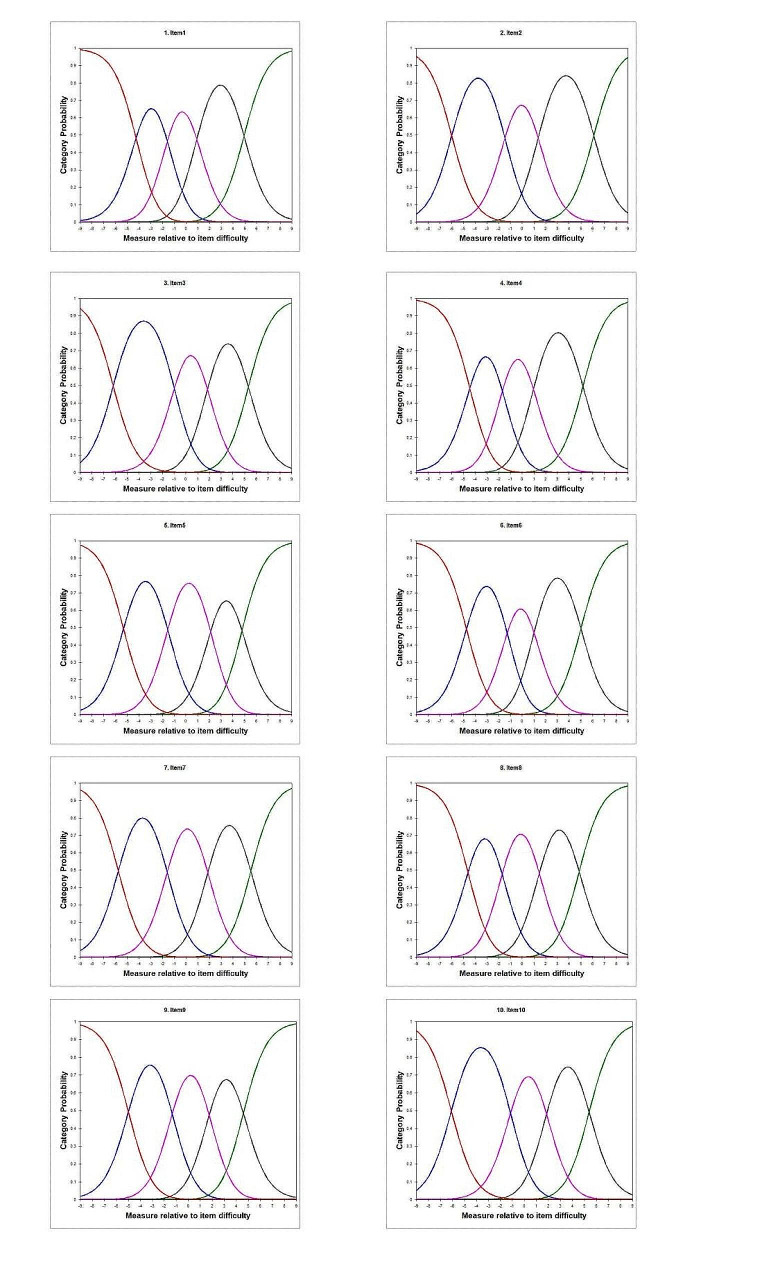
The response characteristic curves of items. The different colors represent the response options. The x axis represented the range of latent trait of item, the y axis “Category Probability” indicated the probability of endorsing a response option (Orange: response category 1, blue: response category 2, rose: response category 3, brown: response category 4, green: response category 5)

Item characteristic curves were shown in Fig. 3, which exhibit the ideal shape, with consistent and distinct peak ordering and appropriate separation of the curves. The assumption of monotonicity was supported. The PCA of the standardized residuals showed that the dimension extracted by the Rasch model accounted for 65.1% of the variance in the data, and the eigenvalue of the unexplained variance in the first extracted component was 2.3. The CSE-CSC scale with ten items satisfied the assumption of unidimensionality. Indication of local dependency between 5 item-pairs (Item 1&7, 5&9, 5&10, 8&9, 9&10) was detected with residual correlations > 0.30 in all 45 item-pairs. In terms of content, the observed local dependency within the scale made sense since these item pairs focused on caregiver confidence in patient symptom management or monitoring.

#### Rasch model fit

The rating scale model showed a good fit to the data. Only one item deviated from the model with both infit and outfit MNSQ were greater than 1.50 (Table 5). All the items produced acceptable point-measure correlations greater than 0.3.

**Table 5:** Rasch model analyses of the CSE-CSC scale

Item	Score (M ± SD)	Measure	Model S.E.	MNSQ		Point-measure Correlation	
				Infit	Outfit	Corr.	Exp.
1	2.88 ± 0.991	1.96	0.09	2.13	2.16	0.59	0.80
5	3.32 ± 0.903	0.56	0.09	0.99	1.01	0.79	0.79
3	3.43 ± 0.902	0.18	0.09	0.97	0.99	0.80	0.79
9	3.45 ± 0.944	0.12	0.09	0.77	0.76	0.87	0.79
10	3.46 ± 0.885	0.09	0.09	0.71	0.71	0.85	0.79
8	3.50 ± 0.907	-0.03	0.09	0.85	0.84	0.83	0.79
7	3.54 ± 0.844	-0.19	0.09	0.80	0.81	0.82	0.79
2	3.73 ± 0.780	-0.83	0.09	0.77	0.78	0.80	0.79
4	3.73 ± 0.850	-0.83	0.09	0.79	0.77	0.83	0.79
6	3.78 ± 0.872	-1.02	0.10	1.10	1.05	0.77	0.78

#### Reliability analysis

The person reliability index (0.93) represented strong consistency among individuals, while the item reliability index (0.99) showed an excellent score. The separation index for individuals in our analysis was 3.51, demonstrating that the scale distinguished the subject population into at least three different ability levels. The separation index of items was 8.36, indicating a clear hierarchy of difficulty among scale items.

#### Item measure and the wright map

**Fig. 4:**
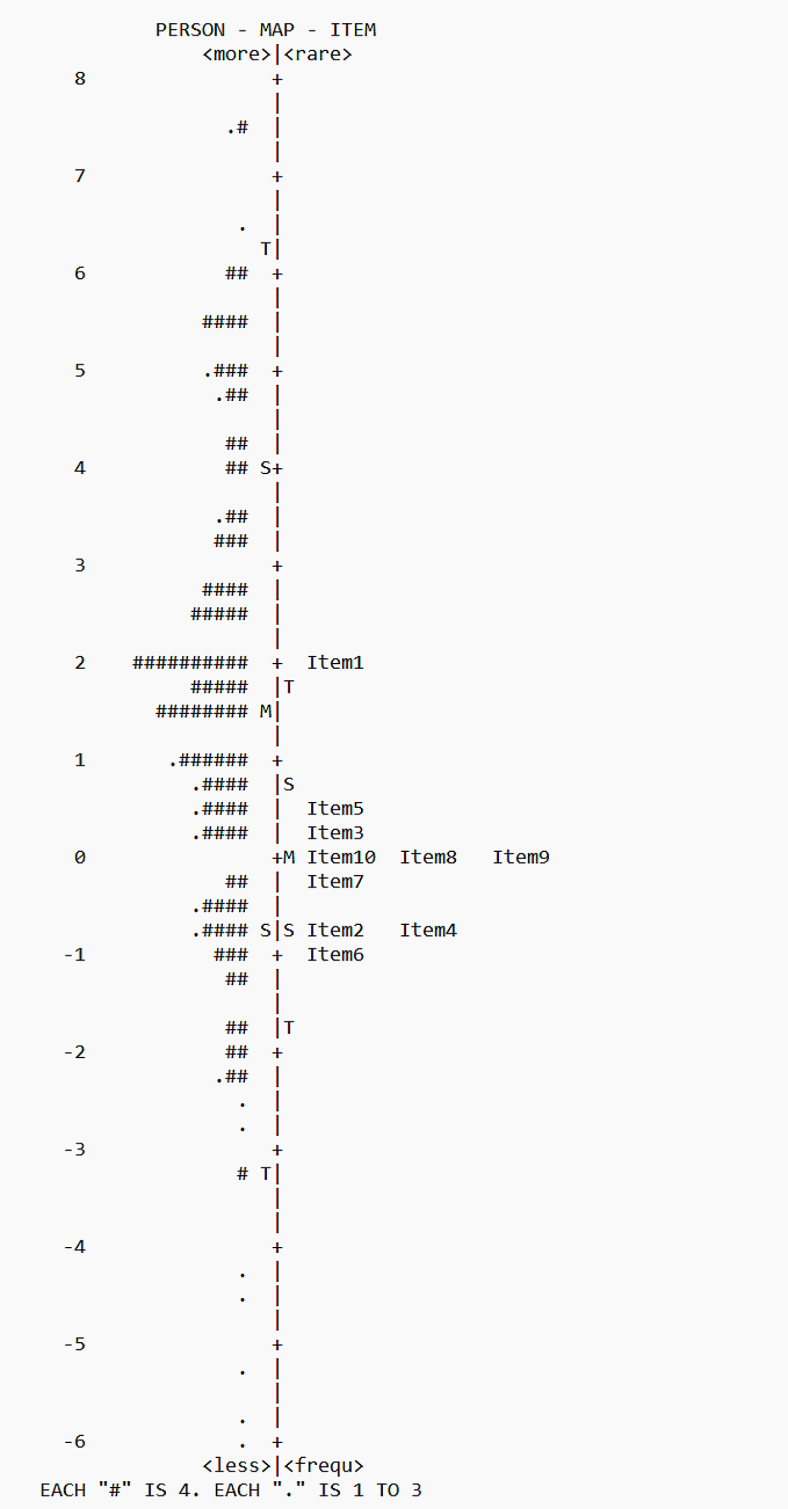
Wright person-item Rasch map for the CSE-CSC scale (*n* = 406). The right side of the figure showed the distribution of items, with the most difficult items at the top to the least difficult at the bottom. On the left side, the distribution represented respondents’ measured abilities, from the most capable at the top to the least capable at the bottom. The symbol of “#” represented 4 persons, and “.” represents 1–3 persons

In this study, participants’ abilities ranged from − 8.41 to 7.50 logits, and the difficulty of items ranged from − 1.02 to 1.91 logits. As illustrated in Table 4, item 1 (‘Keep the illness of the person you care for stable and free of symptoms?’) was identified as the most challenging item (1.96 logits) for all respondents. In contrast, item 6 (‘Recognize symptoms in the person you care for if they occur?’) had the lowest difficulty (-1.02 logits). The Wright map visualization for the CSE-CSC scale was presented in Fig. 4. In this study, the average person’s ability was slightly greater than the average item difficulty, indicating a generally good level of caregiver self-efficacy in contributing to patients’ self-care. The figure also showed that a small subset of individuals had high self-efficacy ability with no corresponding questions to match them. This observation could potentially indicate a ceiling effect in the scale’s IRT analysis [39].

#### Measurement invariance

As shown in Table 6, no items exhibited DIF (prob > 0.05) for the male and female subgroups of respondents. The non-significant result revealed a similar level of self-efficiency in contributing to patient self-care among participants in different genders.

**Table 6:** Differential item functioning (DIF) analysis of the CSE-CSC scale

Item	DIF Measure(± SE)		DIF Contrast (± SE)	Rasch-Welch	
	Male	Female		t	Prob.
1	2.01(± 0.14)	1.93(± 0.11)	0.08(± 0.18)	0.45	0.6514
2	-1.00(± 0.15)	-0.73(± 0.12)	-0.27(± 0.19)	-1.40	0.1623
3	-0.01(± 0.15)	0.30(± 0.11)	-0.31(± 0.19)	-1.64	0.1011
4	-0.89(± 0.15)	-0.80(± 0.12)	-0.08(± 0.19)	-0.43	0.6687
5	0.52(± 0.15)	0.58(± 0.11)	-0.06(± 0.18)	-0.33	0.7441
6	-0.98(± 0.15)	-1.05(± 0.12)	0.07(± 0.20)	0.37	0.7116
7	-0.23(± 0.15)	-0.17(± 0.12)	-0.06(± 0.19)	-0.30	0.7618
8	0.12(± 0.15)	-0.12(± 0.12)	0.24(± 0.19)	1.26	0.2071
9	0.27(± 0.15)	0.02(± 0.12)	0.25(± 0.19)	1.35	0.1777
10	0.16(± 0.15)	0.04(± 0.12)	0.12(± 0.19)	0.63	0.5279

## Discussion

In this study, the CSE-CSC scale was translated and adapted to the Chinese version, following a clear and user-friendly cultural adaptation guideline to ensure cultural appropriateness [42]. From data collected across multiple research centers, CTT and IRT analyses revealed favorable psychometric characteristics, including reliability, validity, difficulty degree, and measurement invariance. The reliable and valid CSE-CSC scale will contribute to a more accurate evaluation and in-depth understanding of caregiver self-efficacy levels among patients with MCCs. Clinical nurses in China can utilize this scale to gain insights into the role of caregiver self-efficacy in patient and caregiver outcomes. Furthermore, the scale provides a new perspective for evaluating intervention programs targeting patients with MCCs, which can help us select more effective health management strategies to address the unique needs of patients with MCCs and improve the overall quality of care.

The reliability and validity of the Chinese version of the CSE-CSC were unquestionable. In CTT, Cronbach’s alpha reflected the intrinsic reliability, and ICC reflected extrinsic reliability. Our study demonstrated that the Chinese version of the CSE-CSC scale exhibited high internal consistency and temporal stability. In Rasch analysis, the ideal person/item reliability and separation index indicated the scale’s ability to distinguish different potential characteristic populations and achieve good measurement precision. In CTT, this study conducted CFA based on the scale structure summarized by the developer and its possible factor structure. As a result, the overall fit index of the first-order 2-factor model surpassed that of the first-order 1-factor and second-order 2-factor models. In IRT, the total scale was evaluated unidimensionally first. Interestingly, the total scale met the assumption of unidimensionality. Although CTT and IRT supported different factor structures, we did not deny the scale’s structural validity and stability due to the different theoretical and hypothesis bases of the two methods. We need more ground to support the existence of two dimensions or to encourage users to use the two dimensions as separate subscales.

Overall, the scale had moderate difficulty and a good differentiating degree, allowing for an accurate reflection of the actual level of caregiver self-efficacy in Chinese patients with MCCs. As observed in the Wright map, individual ability was greater than the difficulty of the scale. However, the overall level of caregiver self-efficacy in China appeared to be lower than that in Italy [20]. Individuals exhibited unstable responses to item 1, which owned the highest difficulty level among all the items. The instability in response may be attributed to the Rasch model’s expectation of high scores while participants performed poorly on this particular item. MCCs mean patients suffer a higher symptom burden and an increased risk of unplanned readmission [43, 44]. In a Canadian qualitative study, caregivers described their experience of caring for a person with MCCs as overwhelming, stressful, and challenging [45]. Caregivers of patients with MCCs may feel isolated and lack confidence in managing complex care tasks and maintaining the stable condition of patients. In our study, items 3, 5, and 7–10 were moderately difficult, while items 2, 4, and 6 were the simplest. Most caregivers lived in urban areas and had experienced secondary school education or higher. Furthermore, 85% of the caregivers had undertaken care tasks for over three years. Their extensive care experience allowed them to be familiar with the patient’s situation and their responsibilities in performing care tasks, such as following the treatment plan, monitoring health conditions, and recognizing changes in health conditions [46]. Furthermore, caregiver self-efficacy had a strong positive correlation with their contribution to self-care maintenance, monitoring, and management. This finding provided an effective breakthrough to promote the caregiver’s contribution to the patient’s self-care.

### Strength and limitation

The psychometric characteristics of the CSE-CSC scale measured in this study were sufficient, and this scale provided a tool with statistical sufficiency and objectivity to measure caregiver self-efficacy in contributing to patient self-care in China. Although conducting a multiple center research trying to balance bias in sample selection, it was essential to acknowledge and consider some limitations. Firstly, the recruitment and testing of subjects were completed in hospital settings in our study. Caregivers in community settings were not considered due to limited energy and resources. Secondly, this study was conducted in four hospitals in Tianjin, China. Since most patients received treatment at nearby hospitals, the sample was geographically limited. The results also confirmed that the proportion of residences in our study subjects was uneven, with only 22.7% of caregivers living in town or the countryside. As a result, a larger survey was recommended to validate the scale in a broader and more representative sample, paying more attention to caregivers in community settings or those in town or countryside.

## Conclusions

The Chinese version of the CSE-CSC scale owned sufficient criterion-related validity, reliability, and objectivity. The CSE-CSC scale could accurately measure the self-efficacy of caregivers who care for patients with MCCs in China. With the increasing prevalence of chronic comorbidity, this scale has become essential for enhancing the well-being of both caregivers and patients with MCCs.

## Data Availability

The datasets used and/or analyzed during the current study are available from the corresponding author on reasonable request.

## Abbreviations

The CSE-CSC scale: The Caregiver Self-Efficacy in Contributing to Patient Self-Care Scale
MCCs: Multiple chronic conditions
CTT: Classical test theory
IRT: Item response theory
CC-SC-CII: The Caregiver Contribution to Self-Care of Chronic Illness Inventory
CVI: Content validity index
I-CVI: Content validity index at the item level
S-CVI: Content validity index at the scale level
CFA: Confirmatory factor analysis
RMSEA: The root mean square error of approximation
SRMR: The standardized root mean square residual
CFI: Comparative Fit Index
TLI: Tucker-Lewis Index
ICC: Intraclass correlation coefficients
DIF: Differential item functioning
